# Eat Well to Fight Obesity… and Save Water: The Water Footprint of Different Diets and Caloric Intake and Its Relationship With Adiposity

**DOI:** 10.3389/fnut.2021.694775

**Published:** 2021-07-01

**Authors:** Mariana Lares-Michel, Fatima Ezzahra Housni, Virginia Gabriela Aguilera Cervantes, Presentación Carrillo, Rosa María Michel Nava, Claudia Llanes Cañedo

**Affiliations:** ^1^Instituto de Investigaciones en Comportamiento Alimentario y Nutrición (IICAN), University Center of the South, University of Guadalajara, Ciudad Guzmán, Mexico; ^2^Instituto Universitario de Investigación del Agua, Universidad de Granada, Granada, Spain; ^3^Departamento de Sistemas y Computación, Tecnológico Nacional de México, Campus Ciudad Guzmán, Ciudad Guzmán, Mexico

**Keywords:** water footprint, diet, dietary patterns, obesity, caloric intake, sustainable diets, healthy diets, Mexico

## Abstract

Water scarcity and excess adiposity are two of the main problems worldwide and in Mexico, which is the most obese country in the world and suffers from water scarcity. Food production represents 90% of a person's water footprint (WF), and healthy diets can lead to less WF than do unhealthy diets related to obesity. We calculated the WF of the diet and caloric intake of adults in Mexico and analyzed its relationship with adiposity. Also, the risk of water expenditure due to adiposity and adherence to dietary recommendations regarding WF of international healthy diets were examined. A Food Consumption Frequency Questionnaire (FCFQ) was applied to 395 adults. Body mass index (BMI), associated with adiposity indicators, was used as a reference for grouping a sample into adiposity levels. The WF was calculated according to the WF Assessment Method, considering correction factors and accounting for water involved in cooking and food washing. Our results showed that the Mexican diet spends 6,056 liters per person per day (L p^−1^d^−1^) and is 55% higher than international healthy diets WF. Consumption of beef, milk, fruits, chicken, and fatty cereals represented 56% of total WF. Strong relations appeared between hypercaloric diets and high WF. Diets of people with excess adiposity generated statistically higher WF with extra expenses of 729 L p^−1^d^−1^ compared with the normal adiposity population. Following nutritional recommendations offers a protective factor in water care, whereas not adhering to these represents a risk up to 93 times greater of water expenditure regarding international healthy diets. Therefore, both for the general population and to regulate obesity, adequate diets can help mitigate the problem of water scarcity.

## Introduction

The excess adiposity that characterizes overweight and obesity is considered a worldwide epidemic (1, 2). This problem has consequences on social, economic, and political sectors; only, in 2014, the global economic impact of obesity was estimated to be US$2.0 trillion or 2.8% of the global gross domestic product, considering its health complications, such as diabetes, hypertension, and some types of cancer (1–5). Furthermore, in the last 15 years, it has been recognized that dietary consumption leading to excess adiposity has a higher environmental impact than healthy dietary consumption (6–8). So obesity has been linked to problems such as climate change (9, 10). Although obesity is a complex alteration that includes psychological, neuronal, and hormonal mechanisms, as well as genetic and epigenetic factors (6, 11) in an understanding of its environmental impacts, it is necessary to consider that its main cause is a positive energy balance, generated by excessive high-caloric diets, with poor-quality nutrients (4, 12). This consumption is made possible by food production that requires high amounts of natural resources, notably water. Water expenditure for agriculture and livestock accounts for 90% of the use of an individual of this resource (13).

The amount of water used to produce each type of food can be measured by the Water Footprint (WF). The WF is defined as the volume of freshwater used in the production of an item, such as the foods that make up the diets of individuals (14). This index has three components (green, blue, and grey), depending on the type of water use: green WF for rainwater used in agriculture; blue WF for irrigation water for agriculture, as well as for livestock drinking and service water; and grey WF for water needed to assimilate pollutants, including agrochemicals dilution and water needed to cook and wash food (15, 16).

The WF index has been used in different parts of the world to evaluate the water needed to supply the diets of some countries. It has been recognized that healthy and plant-based diets, such as vegetarian and Mediterranean, have a lower total WF than other diets that are considered unhealthy such as the western diet, which is rich in fats, sugars, meats, and ultra-processed foods (17–22). In addition, adherence to the dietary recommendations of each country can provide savings of up to 52% of WF (23, 24). However, until today, few studies have evaluated the WF of human diets in a real-life context, and the use of official databases of food consumption or hypothetical scenarios has been the most common method for dietary WF assessments (18, 19, 25).

Despite the information that these studies have provided for research on the WF of diets with different nutritional compositions and in different regions of the world, the relationship between the WF and the diet of people with different degrees of adiposity has not yet been analyzed. That is, no studies are available on the impact of obesity in real-life situations on the WF. In this context, Mexico is an ideal model country, being considered the most obese country in the world (26, 27) while suffering from water scarcity (28).

In 2006, it was reported that more than 12 million Mexicans lacked daily access to water (29), but, in 2018, it was predicted that water availability of 1990 (5,000 m^3^ p^−1^d^−1^) would decrease to <3,000 m^3^ p^−1^d^−1^ by 2050 (28), and, currently, the basins of the country have an estimated deficit of more than 500 million m^3^ of water per year (30).

The diet of Mexicans has been classified as unhealthy and densely caloric. In addition, it is known that the consumption of food by Mexicans deviates far from the dietary recommendations offered in the country (26, 31). This could be one of the main causes of the obesity figures currently available. In 2016, Mexico was reported to be the most obese country in the world, with a combined prevalence of overweight and obesity of 49.4 million people, i.e., 72.5% in the adult population (27, 32). By 2018, these figures raised to 75.2% (26). Food consumption leading to these figures could be related to the high impact on water in Mexico. In this context, we investigated whether a relationship exists between two of the main problems in Mexico: obesity and water scarcity. Although obesity is a sensitive topic that must be aboard from a multidisciplinary perspective, its implications for the environment must be highlighted to propose alternatives to improve both nutritional and environmental issues without stigmatization of this problem. Thus, the objective of this study was to calculate the WF of the diet and caloric intake of adults in Mexico and analyze its relationship with adiposity. Also, the risk of water expenditure due to adiposity and adherence to dietary recommendations regarding WF of international diets were examined.

## Materials and Methods

### Subjects

A quantitative cross-sectional study was performed in a representative sample of Jalisco, Mexico. A total of 509 adult volunteers between 18 and 74 years old of both genders [194 males (38%) and 315 females (62%)] were evaluated. However, as will be explained later, we identified energy intake under-reporters. After that analysis, the sample size was 395 [141 males (36%) and 254 females (64%)]. The sample after analysis of the under-reporters was still representative of Jalisco (33) (minimum of 384 according to the formula as shown in Supplementary Material 1). Data collection was carried out in four urban areas of Jalisco, Mexico (34). The subjects attended nutritional consultation in Guadalajara, Zapopan, Tlajomulco de Zúñiga, and Zapotlán el Grande.

### Body Composition and Anthropometry

As body-composition data on adiposity, body weight, percentage of body fat, and visceral fat were measured with specialized bioelectric impedance equipment (Omron® HBF-511T-E/HBF-511B-E). Waist and hips circumferences were also measured with a Lufkin®s brand metal tape measure. Waist circumference was measured midway between the lower rib and the iliac crest, at the end of normal expiration. The circumference of the hip was measured at the most prominent part of hips. With a base in waist and hips circumferences, the waist-hip ratio was calculated. Height was measured with an ionized aluminum stadiometer Smartmet®. All measurements were performed by certified nutritionists, following the techniques of Suverza and Haua (35).

Based on height and weight, the body mass index (BMI) was calculated by dividing the body weight [in kilograms (kg)] between the height squared (in centimeters [cm]). This indicator was normalized by age in adults over 60 years. For the grouping sample, adiposity was classified as normal or excessive with the use of the WHO (36, 37) classification for BMI, since it has been the most used indicator in epidemiological studies (11) and in studies that evaluate the environmental impact of diets in individuals (19, 38). Individuals with a BMI <25 were considered as normal adiposity. People with a BMI ≥ 25 were classified into the excess adiposity group. In adults over 60 years of age, the excess adiposity group was made up of those persons with a BMI > 28. Adults with BMI ≤ 18.5 and older adults with BMI ≤ 23 were included in the normal adiposity group. Since BMI is a relationship between weight and height and does not directly identify adiposity levels, this index was associated with body-fat percentage, waist circumference, visceral fat, and the waist-hip ratio (11).

### Dietary and Caloric Intake Assessment

A Food-Consumption Frequency Questionnaire (FCFQ) validated for the Mexican population (39) was applied in nutritional consultation by an interview in two residential areas in Zapopan and Tlajomulco de Zuñiga. Data collection by nutritional consultation was also carried out in the Anthropometric Laboratory of the Instituto de Investigaciones en Comportamiento Alimentario y Nutrición (IICAN) at Zapotlán el Grande and in one government dependence of Guadalajara where the nutritional assessment was provided for the working personnel. FCFQ included 162 items, which were subdivided into 184 foods for a more detailed analysis (see Supplementary Material 2). The average grams of food consumption were calculated from the frequency of consumption of the rations established in the FCFQ. The amounts were divided or multiplied as appropriate. The caloric intake was calculated with the use of Mexican food-composition tables (40, 41). For data collection, food replicas, food portions images, measuring cups, and scoops were used. It is important to mention that, since we evaluated the frequency of consumption of foods, and not only the foods ingest in 1 day, the proper term to use is “dietary pattern” (42, 43). However, for comparative purposes with available publications, we are referring to “diet” in this study (18–21, 24, 44, 45).

### Identification of Energy Intake Under-Reporters

Under-reporting has been pointed out as an important problem when evaluating the diet of populations (46) and its environmental impact (47), especially if evaluating people with overweight or obesity (48). Due to this, the reported caloric intake of the population was compared with their energy requirements, as has been suggested by Huang et al. (49) and Howarth et al. (50). Energy requirements were calculated with pre-established formulas, widely used in Mexico, which consider sex, age, weight, height, and physical activity. We specifically used Harris and Benedict formula (51). Physical activity levels were measured with the International Physical Activity Questionnaire (52). The level of physical activity was classified as low (10% of basal energy requirement), moderate (20% of basal energy requirement), and high (30% of basal energy requirement).

We determined as under reporters those participants whose energy intake was <70% than their energy requirements (according to their specific and individualized needs in accordance with their sex, age, weight, height, and physical activity), as this has been reported by studies that identified implausible under-reporters for the evaluation of the environmental impact of diets (47). Over-reporters were not excluded, since overconsumption has been referred to in the Mexican population as a real problem, which, indeed, is the main cause of overweight and obesity problems (26, 31).

### Calculation of the Water Footprint

To assess the WF of the diet, we followed the proposed methodology by Lares-Michel et al. (16), which follows the WF assessment methodology (15). Therefore, we adhered to the three basic steps for dietary WF calculation: (1) we evaluated the diet through a nationally validated questionnaire, which was applied by nutrition experts; (2) the WF of each food was calculated based on WF country/state-specific databases. Accordingly, we used the tables for Jalisco (the area of study) for crops (53, 54) and the national databases of Mexico for livestock (55, 56). When data of Mexico were not available, we used international tables (57, 58). In all calculations, the corresponding correction factors were applied for converting cooked and processed (i.e., peeled, without seeds) foods to raw or/and unprocessed foods. Also, we accounted for water involved in cooking and washing food (16); (3) we calculated the WF of multi-ingredient dishes, using reported tables for Mexico or calculating ingredients through interviews during FCFQ application or reviewing nutritional labels (16). The WF was specifically calculated per person per day (L p^−1^d^−1^) as defined in the Global WF Standard (15).

Foods were classified into 22 groups according to their nutritional composition or WF. Foods of animal origin were more accurately classified according to WF. These foods included milk and yogurt, cheeses, eggs, chicken, beef, pork, processed meats, lamb, fish, and seafood. Those of vegetable origin were classified according to the Mexican-equivalent food system (41), which is the basis of the food guides in Mexico for food classification (59). This category included vegetables, fruits, and legumes, as well as cereals, which, in turn, were classified as fat-free cereals and cereals with fat. The former included cereals that share a similar nutritional composition, such as corn tortillas, rice, wheat bread, oatmeal, pasta, and tubers, such as potatoes. The classification of cereals with fat included industrialized cereals in which were added to fat or, in certain cases, sugars, for example, pizza, cookies, and cakes. This classification of the Mexican-equivalent food system also includes fat tubers, such as French fries and chips (41).

Concerning to fats, they were classified into fats or oils with and without protein according to Pérez Lizaur et al. (41). Protein fats included nuts, almonds, and peanuts, among others. In the case of oils without protein, oils of different types were included, such as olive, corn, sunflower, and safflower oils. This category also included vegetable fats, such as avocados. Sugars were classified into sugars with and without fat. Those without fat correspond to sugar, sweets, and honey, among others. Within sugars with fat were foods that contain high amounts of sugars and also fats, such as ice cream and chocolates, which, in addition to sharing similar nutritional composition, share similar WF, since their ingredients tend to be similar. Natural and industrialized fruit juices, as well as coffee and tea, soft drinks, and alcoholic beverages, were considered as separate groups, according to their WF (16, 41, 53, 54, 57). The detailed classification of food groups is shown in Supplementary Material 2.

### Water Footprint Variations According to Adherence to Dietary Recommendations and Adiposity

#### Healthy Diets Water Footprint

**Figure 1:**
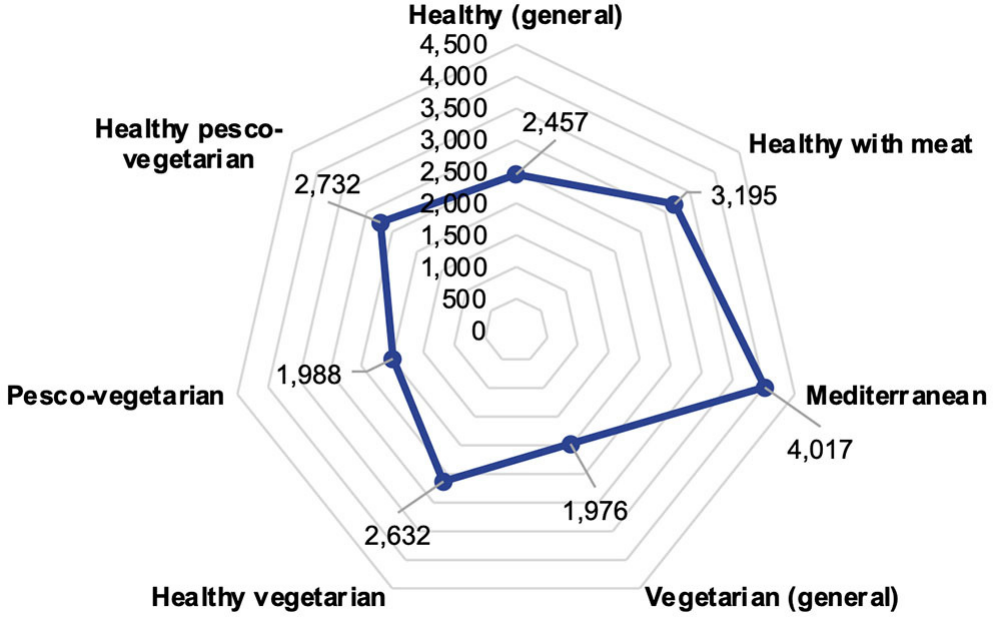
Average green and blue water footprint (WF) of the identified healthy diets in liters per person per day (L p^−1^d^−1^). Average healthy diet WF was 2,714 L p^−1^d^−1^. Detailed information is available in Supplementary Material 3.

For the risk analysis of water expenditure, the WF of healthy diets reported in different countries/regions was reviewed for setting an average WF as a comparison point. Through literature search, we identified seven healthy diets to which their WF has been calculated. This included healthy diets in general (20, 21, 45), healthy with meat included (24), healthy vegetarian (21, 45), and healthy pesco-vegetarian (24); vegetarian (19–21, 45) and pesco-vegetarian (45) were also included, as well as the Mediterranean diet (18, 44). The selected diets had a range from 1,976 L p^−1^d^−1^ to 4,017 L p^−1^d^−1^ (Figure 1). The average WF of these healthy diets was 2,714 L p^−1^d^−1^, which was defined as a cutoff point to determine exposed and unexposed populations in the odds ratio analysis. Since most available studies have just reported green and blue WF, grey WF was excluded from this analysis. The details of this analysis are shown in Supplementary Material 3, and the characteristics of each diet are present in Supplementary Material 4.

#### Water Footprint According to Adiposity

For the generation of cases and controls in odds ratio analysis regarding adiposity, we used BMI, body-fat percentage, waist circumference, visceral fat, and waist–circumference ratio (36, 37, 60–62). Individuals with a BMI equal to or above 25 were considered as cases, and people with a BMI below this figure as controls. In adults over 60 years of age, cases were determined in the population with a BMI >28. Adults with BMI ≤ 18.5–24.9 and older adults with a BMI ≤ 23–27.9 were considered into the control group (36, 37). Cases of excess body fat were determined to be >22% in men and >32% in women. Values under those specified were considered controls (60). A waist circumference above 80 cm in women and 90 cm in men was used to determine cases when these figures were equal or exceeded, and controls when subjects had a waist circumference less than those indicated above (62). Cases and controls regarding visceral fat were determined with the manuals of the Omron® scale, when the level was above or below 9, respectively. In the waist–hips ratio, men with values under 0.90 and women with values under 0.85 were considered controls (61). In these last four indicators, no differences by age are established.

#### Water Footprint Variations Due to Adherence to Dietary Recommendations

For the calculation of risk due to adherence to dietary recommendations, those in force for Mexico were used. Recommendations for the consumption of red meat, including beef, pork, lamb, and processed meats, were considered (59). The recommendations of Macedo-Ojeda et al. (63) for fruits, vegetables, legumes, and fat-free cereals intake were also taken into account. The amounts of consumption recommended by the Ministry of Health of Mexico (64) were used to determine the suggested intake rations of milk, yogurt, cheeses, chicken, eggs, fish, and nuts.

The suggested maximum intake of cereals with fat and sugars with and without fat was also considered (59). In addition, recommendations for drinks, such as soft drinks, natural and industrialized fruit juices, and alcoholic beverages (65), were also included. The recommended daily rations in grams were calculated according to the frequency and suggested amounts of intake.

For the selection of cases and controls regarding adherence to dietary recommendations, cases were considered for all those subjects that exceeded the daily recommended rations of red meat, milk, yogurt, cheese, chicken, eggs, fatty cereals, sugars with and without fat, soft drinks, natural and industrialized fruit juices, and alcoholic beverages. In the situation of fat-free cereals, fruits, vegetables, legumes, fish, and nuts, the subjects whose intake was below those recommended were considered cases. The portions of each food group are detailed in Table 1.

**Table 1:** Compilation of Mexican daily dietary recommendations by food group.

**Food group**	**Recommended amount**	**Unit of measurement**	**References**
Red meat	≤71.42	g p^−1^ d^−1^	(59)
Cereals without fat	≥200	g p^−1^ d^−1^	(63)
Fruits and vegetables	≥400	g p^−1^ d^−1^	(63)
Legumes	≥60	g p^−1^ d^−1^	(63)
Milk and yogurt	≤240	mL p^−1^ d^−1^	(64)
Cheeses	≤40	g p^−1^ d^−1^	(64)
Poultry and eggs	≤56.25	g p^−1^ d^−1^	(64)
Nuts	≥7.28	g p^−1^ d^−1^	(64)
Fish	≥25.71	g p^−1^ d^−1^	(64)
CFS, FS, and NFS	≤10%^a^	kcal p^−1^ d^−1^	(59)
Soft drinks	≤34.28	mL p^−1^ d^−1^	(65)
Natural and industrialized fruit juice	≤125	mL p^−1^ d^−1^	(65)
Alcoholic drinks	≤45	mL p^−1^ d^−1^	(65)

*CFS, Cereals with fat and/or sugars; FS, Fatty sugars; NFS, Not fatty sugars*.

a *Of daily caloric intake*.

### Statistical Analysis

The distribution of the data was verified with the Kolmogorov Smirnoff test. Also, the multicollinearity and heteroscedasticity of the data were analyzed. Following, descriptive analyzes were carried out, reporting means ± standard deviations (SD), medians, minimums (min), and maximums (max) values. The differences between groups were analyzed with the *U* de Mann–Whitney test or with the Chi-squared test for categorical variables. Simple and multiple linear regression models were performed to analyze the association between caloric intake and adiposity indicators with individual dietary WF. Additionally, Spearman correlations were performed between the WF and the adiposity indices.

Likewise, bivariate logistic regressions reporting odds ratios were performed to assess the risk of water expenditure regarding adiposity and adherence to dietary recommendations concerning WF of an international healthy diet. The aforementioned adiposity classifications were used to determine cases and controls in regard to BMI, body-fat percentage, waist circumference, visceral fat, and waist-circumference ratio (36, 37, 60–62). For the selection of cases and controls regarding adherence to dietary recommendations, we used the above-mentioned minimum and maximum daily recommended rations shown in Table 1. To determine the population exposed and not exposed to excessive water expenditure, the average WF of healthy diets was used (Figure 1). Overcoming that WF was considered as being exposed. The analyses were carried out with the STATA/SE 12.0® statistical software.

### Ethics Considerations

This study was approved by the Ethics Committee of the University of Guadalajara CEICUC (registration number CEICUC-PGE-004). Likewise, the principles of the Helsinki declaration were followed, and all the participants were adults who signed informed consent before being included in the study.

## Results

### Sample Characteristics

The demographic and body composition characteristics of the sample are shown in Table 2. The average age was 33.55 ± 15.57 years, and 64.30% were the female population. Average BMI indicated the population is overweight. However, by mean body-fat percentage, males and females were classified as obese. Average waist circumference also indicates abdominal obesity in both genders. Mean visceral fat was only excessive in males, and the average waist-hips ratio was normal in both genders. All adiposity variables were positive associated with BMI.

**Table 2:** Demographic and body composition characteristics and correlational analysis of total sample data in regard to adiposity indicators.

**Characteristics**	**Total sample**	***rho***	***p*-value**	**Male**	**Female**	***p*-value**
	***n* (%)**			***n* (%)**	***n* (%)**	
Total sample	395 (100)	-	-	141 (35.70)	254 (64.30)	** <0.001^b^**
Sex		-	-			
Male	141 (35.70)	-	-	-	-	-
Female	254 (64.30)	-	-	-	-	-
Age	33.55 ± 15.57	-	-	35.35 ± 16.23	32.56 ± 15.14	**0.043^c^**
Weight (kg)	70.44 ± 14.96	-	-	80.58 ± 13.04	64.81 ± 12.85	** <0.001^c^**
Height (cm)	164.55 ± 8.87	-	-	172.53 ± 6.75	160.12 ± 6.48	** <0.001^c^**
BMI (kg m^−2^)	25.91 ± 4.58	-	-	26.97 ± 3.7	25.33 ± 4.91	** <0.001^c^**
BF (%)	34.07 ± 8.76	0.522^a^	** <0.001^a^**	27.8 ± 6.89	37.55 ± 7.69	** <0.001^c^**
WC (cm)	85.34 ± 12.63	0.859^a^	** <0.001^a^**	92.32 ± 11.59	81.47 ± 11.49	** <0.001^c^**
VF (kg)	8.40 ± 3.97	0.736^a^	** <0.001^a^**	9.69 ± 4.17	7.69 ± 3.67	** <0.001^c^**
WHR	0.84 ± 0.08	0.542^a^	** <0.001^a^**	0.89 ± 0.07	0.80 ± 0.06	** <0.001^c^**
HC (cm)	101.66 ± 8.77	-	-	102.72 ± 6.86	101.08 ± 9.63	**0.005^c^**
MM (kg)	27.21 ± 6.30	-	-	33.22 ± 4.56	23.87 ± 4.36	** <0.001^c^**

*BMI, Body mass index; BF, body fat percentage; WC, waist circumference; VF, visceral fat; WHR, waist-hip ratio; HC, Hip's circumference; MM, muscle mass*.

*Adiposity indicators: BF, WC, VF, WHR regarding BMI*.

a *Spearman correlation test between BMI and adiposity indicators*.

b *Chi-squared test*.

c *U de Mann–Whitney test*.

*Statistical significance was considered at p ≤ 0.05 and is shown in bold*.

*A confidence interval at 95%*.

### Water Footprint of the Diet and Caloric Intake

Table 3 shows the average food consumed in the diet of the sample as well as the reported caloric intake, which overpassed 2,415 calories per person per day (kcal p^−1^d^−1^), with a variation of more than 897 kcal p^−1^d^−1^. This consumption generated an average total WF of 6,056, which presented a considerable high standard deviation (more than 2,719 L p^−1^d^−1^) and maximum values that overpassed 18,400 L p^−1^d^−1^, which is related to the type and quantities of foods consumed, as will be explained later. The identified median was 5,439 L p^−1^d^−1^. The green WF represented 83.22% of the total WF of the diet, blue WF represented 8.98%, and grey WF contributed the lowest proportion with 7.78%.

**Table 3:** Dietary and caloric intake and its water footprint (WF).

**Indicator**	**Total sample**		
	**Mean ± SD**	**Median**	**Min – max**
Food intake (g p^−1^d^−1^)	2,378.76 ± 885.47	2,257.17	920.87 – 7,299.18
Caloric intake (Kcal p^−1^d^−1^)	2,415.44 ± 897.62	2,225.00	1,203.62 – 8,803.01
Total WF (L p^−1^d^−1^)	6,055.89 ± 2,719.25	5,439.00	2,103.88 – 18,481.17
Green (L p^−1^d^−1^)	4,952.51 ± 2,287.08	4,449.74	1,646.92 – 14,840.68
Blue (L p^−1^d^−1^)	534.88 ± 217.54	479.05	225.35 – 1,992.68
Grey (L p^−1^d^−1^)	463.2 ± 192.01	410.29	212.52 – 1,873.75

**Figure 2:**
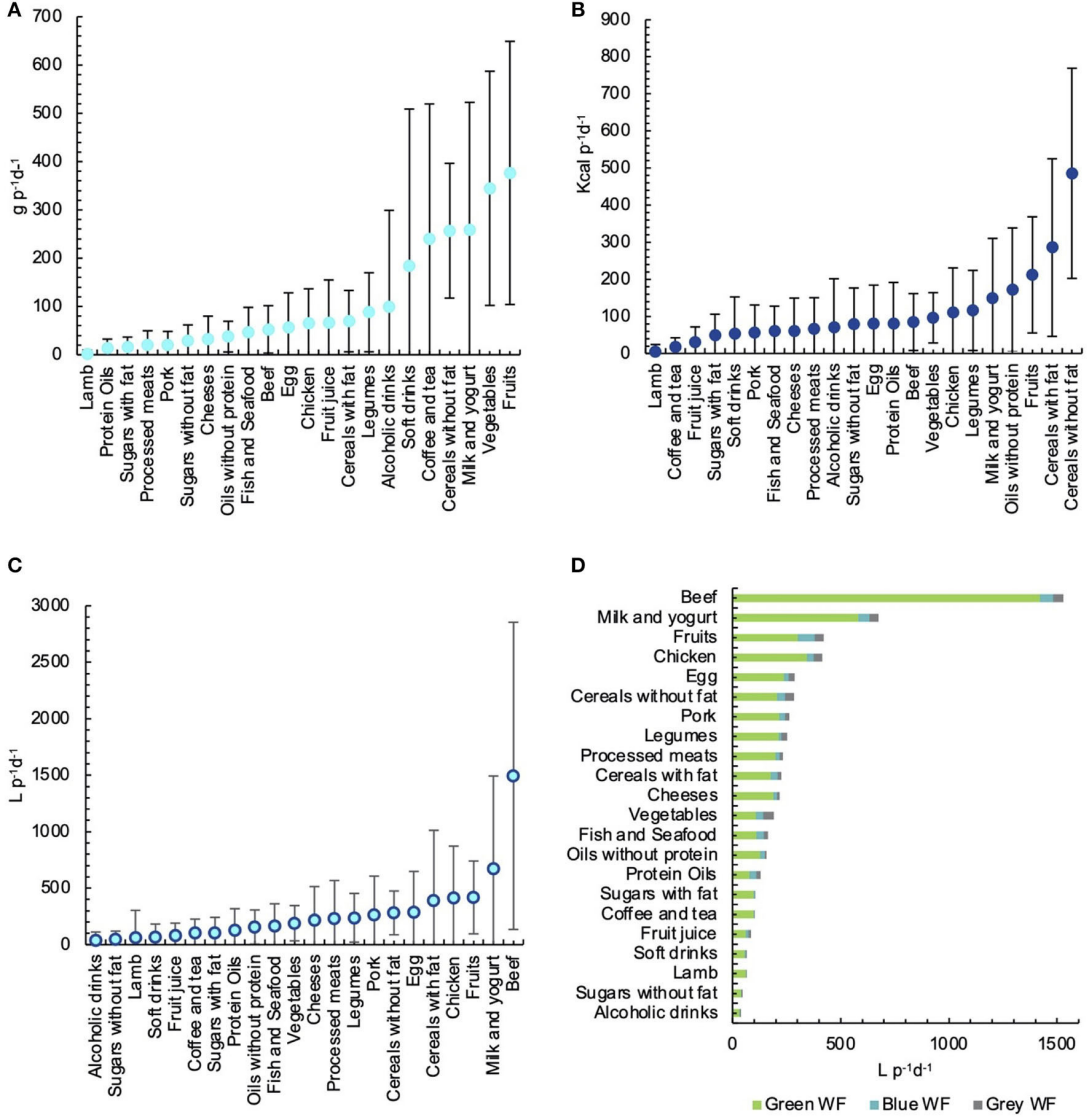
Dietary and caloric intake and green, blue, grey, and total water footprint (WF) of the Mexican diet. **(A)** Grams consumed. **(B)** Calories consumed. **(C)** Total water footprint (WF) of the diet. **(D)** Green, blue, and grey water footprint (WF).

As shown in Figure 2A, the most consumed food group was fruits, representing 15.83% of total food intake, followed by vegetables, which represented 14.49% of total food intake, and milk and yogurt that accounted for 10.88% from total food intake. However, non-fat cereals, such as corn tortillas, bread, and rice, represented the highest caloric intake with 20.11% of total energy intake, followed by cereals with fat, such as pizza, industrialized bread with sugars, and French fries with 11.82% of total energy intake, and fruits, which represented 8.76% of total energy intake (Figure 2B). The first cereals represented only 4.64% of the total WF, while milk and yogurt accounted for 11.13%, fruits for 6.90%, chicken for 6.85%, and cereals with fat accounted for 6.48%. However, beef was the food with the highest WF, contributing to 24.67% of the total WF (Figure 2C).

In general, WF components were equally distributed in all food groups, being the green WF the one with the highest proportion regarding total WF, reaching 97.32% in the lamb case, 93.88% in coffee and tea, 92.41% in sugars with fat, and 92.96% in the beef case. The lowest green WF was found in vegetables with 57.20% and protein oils with 60.86%. Blue WF was the second-highest contributor to total WF, except in the cases of chicken, eggs, legumes, cereals without fat, vegetables, coffee and tea, and fruit juice, where grey WF was higher than blue. The lowest proportion of blue WF was found in beef with 4.12%. From all blue WF of food groups, vegetables, fruits, fish and seafood, and protein oils were the food groups with the highest proportion of blue WF with 17.66, 18.64, 21.00, and 23.86%, respectively. In the case of grey WF, vegetables, fruit juice, protein oils, and cereals without fat were the food groups with the highest proportion of grey WF, with 25.15, 15.56, 15.28, and 14.70%, respectively (Figure 2D).

As shown in the model of Table 4, dietary intake is highly related to total WF (*R*^2^ = 0.945, *p* < 0.001), especially beef, lamb, pork, and processed meats. In all cases, statistical significance was found, except in sugars without fat, sugars with fat, coffee and tea, soft drinks, and alcoholic drinks.

**Table 4:** Multiple linear regression model between water footprint (WF) and dietary intake by food groups.

				***R*****-squared** **=** **0.945**	
				**Adj** ***R*****-squared** **=** **0.942**	
				***p*** **<** **0.001**	
	**β**	**Std. Err**.	***t***	***p*-value**	**[95% CI]**
Milk and yogurt	2.87	0.13	22.42	** <0.000**	2.62 – 3.13
Cheeses	5.76	0.78	7.42	** <0.000**	4.23 – 7.28
Egg	4.22	0.53	7.95	** <0.000**	3.17 – 5.26
Chicken	6.35	0.52	12.28	** <0.000**	5.33 – 7.37
Beef	29.40	0.83	35.35	** <0.000**	27.77 – 31.04
Pork	11.66	1.34	8.73	** <0.000**	9.03 – 14.29
Processed meats	12.44	1.25	9.96	** <0.000**	9.98 – 14.9
Lamb	32.01	4.59	6.97	** <0.000**	22.98 – 41.04
Fish and Seafood	2.83	0.76	3.71	** <0.000**	1.33 – 4.33
Vegetables	0.73	0.16	4.56	** <0.000**	0.42 – 1.04
Fruits	0.78	0.15	5.14	** <0.000**	0.48 – 1.08
Legumes	4.49	0.46	9.86	** <0.000**	3.59 – 5.38
Cereals without fat	0.64	0.28	2.29	**0.023**	0.09 – 1.18
Cereals with fat	6.68	0.60	11.07	** <0.000**	5.5 – 7.87
Oils without protein	3.88	1.19	3.27	** <0.001**	1.55 – 6.22
Protein oils	10.11	1.91	5.29	** <0.000**	6.35 – 13.87
Sugars without fat	0.00	1.09	0.00	0.997	−2.15 – 2.15
Sugars with fat	1.78	1.89	0.94	0.348	−1.94 – 5.5
Fruit juice	0.93	0.40	2.33	**0.020**	0.15 – 1.72
Coffee and tea	0.06	0.14	0.43	0.665	−0.21 – 0.33
Soft drinks	0.20	0.11	1.90	0.058	−0.01 – 0.41
Alcoholic drinks	0.20	0.17	1.17	0.242	−0.14 – 0.54

*Statistical significance was considered at p ≤ 0.05 and is in bold*.

*A confidence interval at 95%*.

**Figure 3:**
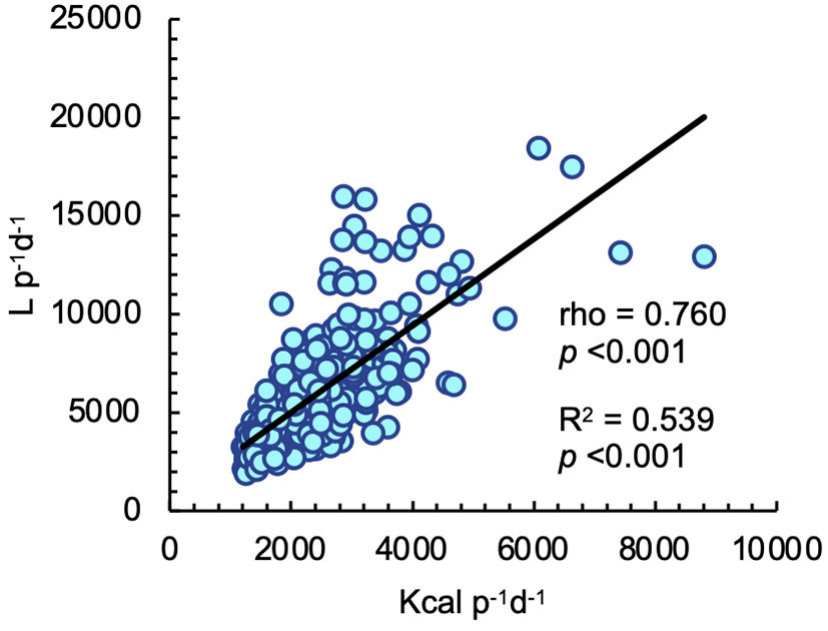
Simple linear regression model between caloric intake and dietary total water footprint (WF). Statistical significance was considered at *p* ≤ 0.05. *rho* was obtained from spearman correlation.

In regard of caloric intake, a strong and significant association between total caloric intake and WF was identified (*rho* = 0.760, *p* < 0.001), which resulted in a moderate relationship (*R*^2^ = 0.539, *p* < 0.001) (Figure 3).

**Figure 4:**
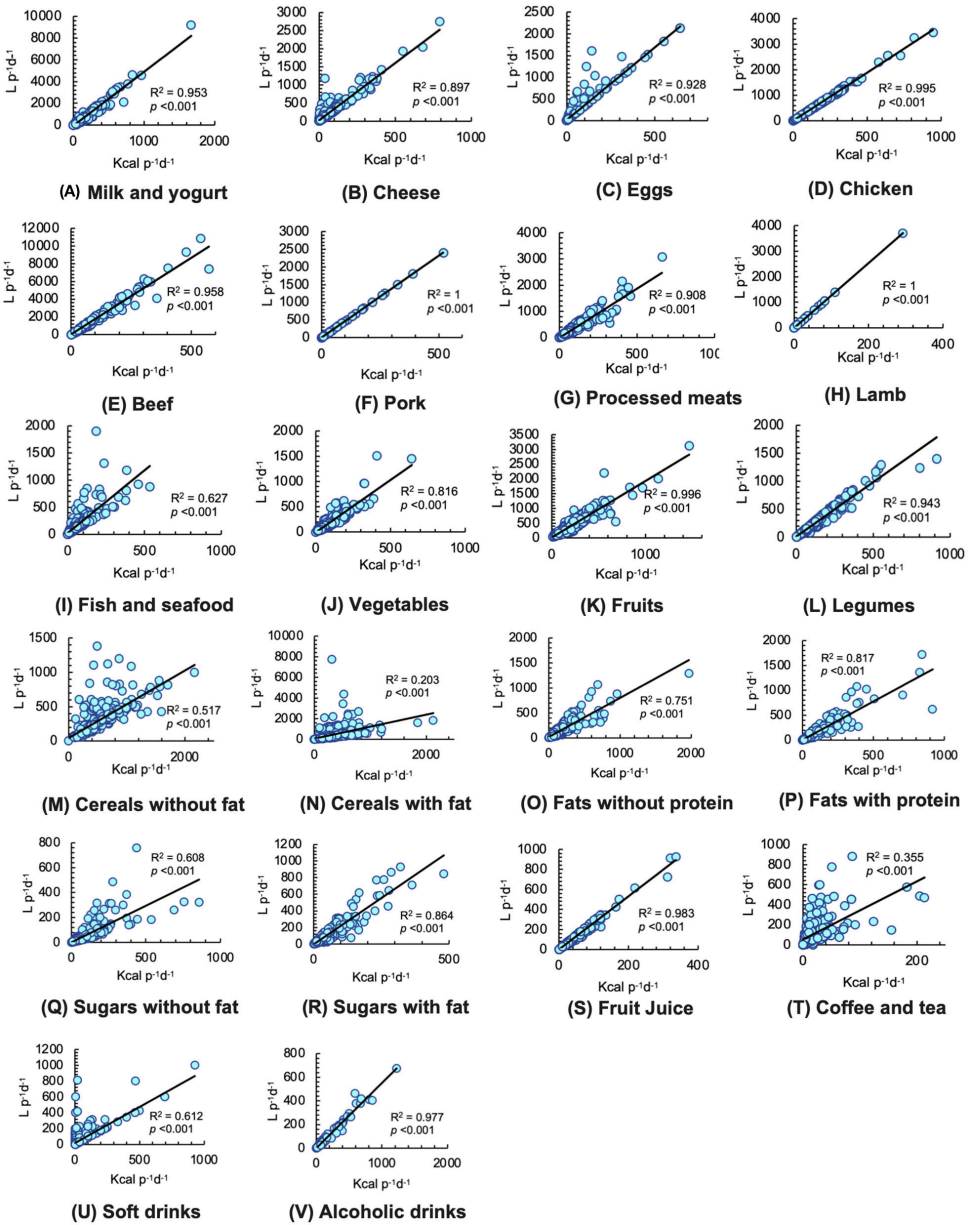
Simple linear regressions model between caloric intake and dietary total water footprint (WF) by food groups. Statistical significance was considered at *p* ≤ 0.05. **(A)** Milk and yogurt. **(B)** Cheese. **(C)** Eggs. **(D)** Chicken. **(E)** Beef. **(F)** Pork. **(G)** Processed meats. **(H)** Lamb. **(I)** Fish and seafood. **(J)** Vegetables. **(K)** Fruits. **(L)** Legumes. **(M)** Cereals without fat. **(N)** Cereals with fat. **(O)** Fats without protein. **(P)** Fats with protein. **(Q)** Sugars without fat. **(R)** Sugars with fat. **(S)** Fruit juice. **(T)** Coffee and tea. **(U)** Soft drinks. **(V)** Alcoholic drinks.

In all food groups, significant and positive relationships were identified, in particular, *R*^2^ values of 1 were found in the cases of pork (Figure 4F) (*p* < 0.001) and lamb (Figure 4H) (*p* < 0.001). In addition, in the case of milk and yogurt (Figure 4A), cheese (Figure 4B), eggs (Figure 4C), chicken (Figure 4D), beef (Figure 4E), processed meats (Figure 4G), vegetables (Figure 4J), fruits (Figure 4K), legumes (Figure 4L), fats with protein (Figure 4P), sugars with fat (Figure 4R), fruit juice (Figure 4S), and alcoholic drinks (Figure 4V), *R*^2^ values between 0.81 and 0.99 (*p* < 0.001) were identified. Despite being high-calorie foods, cereals with fat (Figure 4N) were the food group with the lowest values (*R*^2^ =0.203, *p* < 0.001). Also, coefficients of determination of fish and seafood (Figure 4I), cereals without fat (Figure 4M), fats without protein (Figure 4O), sugars without fat (Figure 4Q), coffee and tea (Figure 4T), and soft drinks (Figure 4U) were between 0.35 and 0.75 (*p* < 0.001).

### Water Footprint Variation as Function of Adiposity

The excess adiposity group was made up of 51.90% of the sample. Women predominated in both groups. The average age was higher in the excess adiposity group. All body composition data were statistically different in both groups, except for muscle mass (Table 5).

**Table 5:** Comparative analysis of demographic and body composition characteristics by adiposity groups.

**Characteristics**	**Normal adiposity**	**Excess adiposity**	***p*-value**
	***n* (%)**	***n* (%)**	
Total sample	190 (48.10)	205 (51.90)	** <0.001^a^**
**Sex**			
Male	51 (26.84)	90 (43.91)	** <0.001^b^**
Female	139 (73.15)	115 (56.09)	** <0.001^b^**
Age	29.59 ± 14.72	37.22 ± 15.48	** <0.001^b^**
Weight (kg)	59.98 ± 8.90	80.13 ± 12.74	** <0.001^b^**
Height (cm)	163.37 ± 7.67	165.65 ± 9.74	**0.016^b^**
BMI (kg m^−2^)	22.30 ± 2.07	29.26 ± 3.62	** <0.001^b^**
BF (%)	29.91 ± 7.16	37.93 ± 8.34	** <0.001^b^**
WC (cm)	76.62 ± 8.36	93.42 ± 10.35	** <0.001^b^**
VF (kg)	6.01 ± 2.67	10.62 ± 3.68	** <0.001^b^**
WHR	0.80 ± 0.07	0.87 ± 0.08	** <0.001^b^**
HC (cm)	95.62 ± 5.32	107.27 ± 7.53	** <0.001^b^**
MM (kg)	26.77 ± 6.78	27.61 ± 5.81	0.254^b^

*BMI, Body mass index; BF, body fat percentage; WC, waist circumference; VF, visceral fat; WHR, waist-hip ratio; HC, Hip's circumference; MM, muscle mass*.

*Adiposity indicators: BF, WC, VF, WHR regarding BMI*.

a *Chi-squared test*.

b *U de Mann-Whitney test*.

*Statistical significance was considered at p ≤ 0.05 and is in bold*.

*A confidence interval at 95%*.

As presented in Table 6, total food (*p* < 0.001) and caloric intake (*p* = 0.008), as well as total WF (*p* = 0.011), green (*p* = 0.011), blue (*p* = 0.034), and grey (*p* = 0.023) components, were higher in the excess adiposity group. There were found differences of more than 233 kcal p^−1^d^−1^ between groups. Regarding total WF, a difference of 728.86 L p^−1^d^−1^ was identified between groups. Median was also higher in the excess adiposity group. Green WF was the highest component in both groups, followed by blue WF and grey WF. It is important to note that standard deviations found were considerably high, representing almost 50% of total WF, which is principally related to the type and the amount of foods consumed, being the animal origin products the principal responsible for the variations found, since persons consuming more meat reached total WFs over 14,014 L p^−1^d^−1^ in the group with normal adiposity and more than 18,481 L p^−1^d^−1^ in the group with excess adiposity.

**Table 6:** Dietary and caloric intake and its water footprint (WF) by adiposity groups.

**Indicator**	**Normal adiposity**			**Excess adiposity**			***p*-value**
	**Mean ± SD**	**Median**	**Min – max**	**Mean ± SD**	**Median**	**Min – max**	
Food intake (g p^−1^d^−1^)	2,190.06 ± 832.86	2,067.23	920.87 – 7,299.18	2,553.66 ± 898.75	2,369.63	1,151.2 – 6,844.66	** <0.001^a^**
Caloric intake (Kcal p^−1^d^−1^)	2,294.05 ± 798.6	2,097.09	1,203.62 – 7,647.41	2,527.94 ± 968.94	2,304.34	1,292.31 – 8,803.01	**0.008^a^**
Total WF (L p^−1^d^−1^)	5,677.62 ± 2,336.28	5,159.07	2,103.88 – 14,014.26	6,406.48 ± 2,994.58	5,624.74	2,376.57 – 18,481.17	**0.011^a^**
Green (L p^−1^d^−1^)	4,638.94 ± 2,018.93	4,103.08	1,646.92 – 11,829.45	5,243.14 ± 2,479.86	4,668.10	1,883.55 – 14,840.68	**0.006^a^**
Blue (L p^−1^d^−1^)	509.75 ± 189.91	458.14	239.46 – 1,224.6	558.16 ± 238.45	494.72	225.35 – 1,992.68	**0.034^a^**
Grey (L p^−1^d^−1^)	442.68 ± 169.52	389.65	212.52 – 1,055.02	482.22 ± 209.35	434.01	215.96 – 1,873.75	**0.023^a^**

a *U de Mann–Whitney test*.

*Statistical significance was considered at p ≤ 0.05 and is in bold*.

*A confidence interval at 95%*.

**Figure 5:**
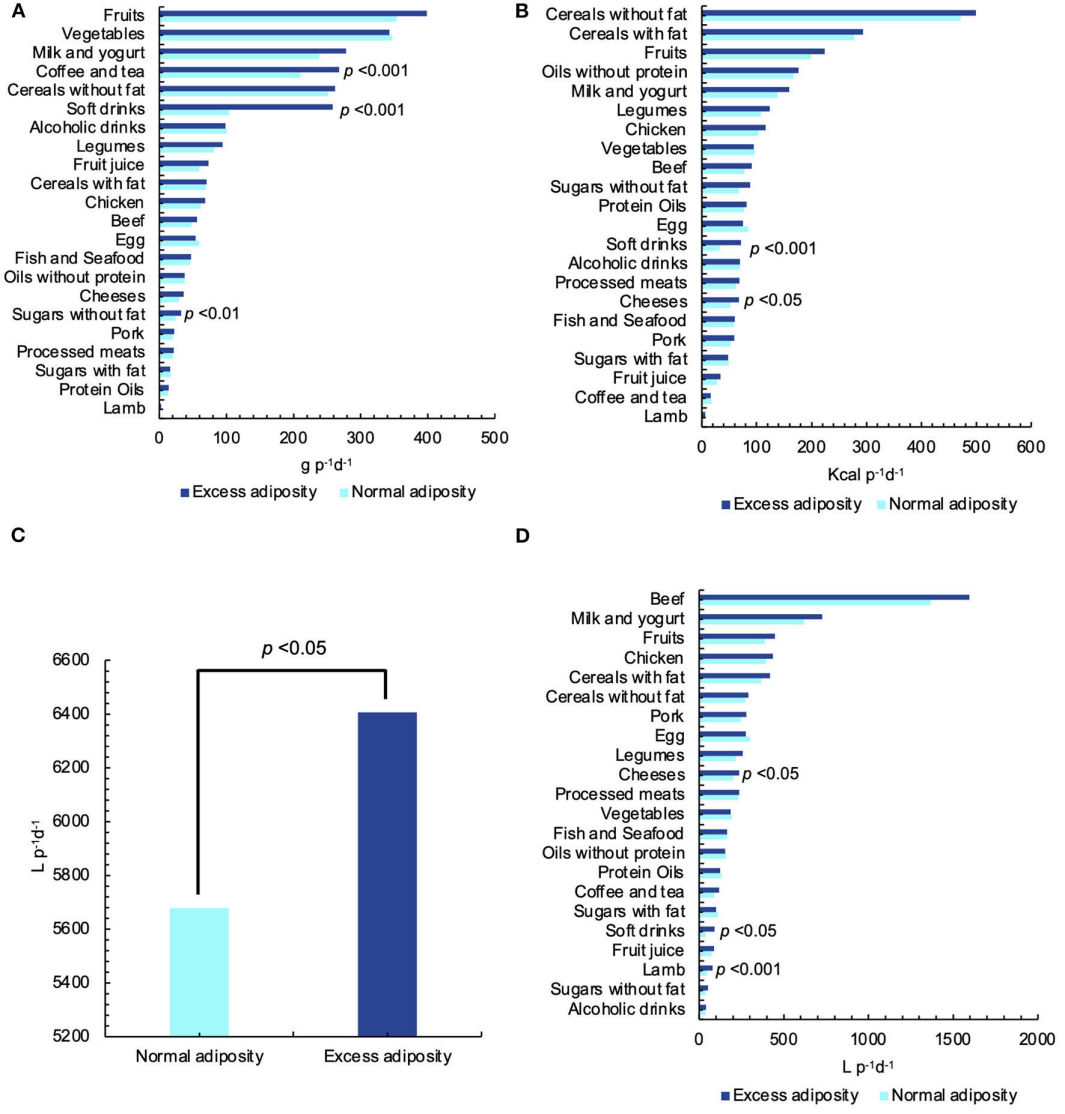
Food and caloric intake and its water footprint (WF) by food groups in the adiposity groups. Statistical significance was considered at *p* ≤ 0.05. **(A)** Food groups consumed by adiposity groups. **(B)** Caloric intake by adiposity groups. **(C)** Total water footprint (WF) by adiposity groups. **(D)** Total water footprint (WF) of food groups by adiposity groups.

As can be seen in Figure 5A, the dietary intake of the excess adiposity group resulted higher in almost all the food groups, especially in coffee and tea consumption (*p* < 0.005), soft drinks intake (*p* < 0.001), and sugars without fat consumption (*p* < 0.01). The only food groups where a trend of higher intake in the normal adiposity group was found are vegetables (*p* = 0.592), alcoholic drinks (*p* = 0.286), eggs (*p* = 0.196), oils without protein (*p* = 0.970), and sugars with fat (*p* = 0.189). However, the differences were not statistically significant.

The caloric intake from soft drinks (*p* < 0.001) and calories of cheese intake (*p* < 0.05) were higher in the excess adiposity group. In almost all food groups, energy intake was higher in the excess adiposity group, except for vegetables (*p* = 0.470), eggs (*p* = 0.111), sugar with fat (*p* = 0.242), and coffee and tea (*p* = 0.826) (Figure 5B).

As mentioned before, the total WF of the diet of adiposity groups was statistically different (*p* < 0.05). The normal adiposity group presented a total WF 13% lower than the group with excess adiposity (Figure 5C). WF components were similar in both groups, only blue and grey WFs were slightly higher in the normal adiposity group (9.12 and 7.91%, respectively) in comparison with the excess adiposity group (8.88 and 7.67%, respectively). Green WF was higher in the excess adiposity group (83.44 vs. 82.97%) (Table 6).

Regarding total WF by food groups, in Figure 5D, it is possible to observe that the total WF of cheeses (*p* < 0.05), soft drinks (*p* < 0.05), and lamb (*p* < 0.05) was higher in the excess adiposity group. In almost all the food groups, the WF of the excess adiposity group was higher, reaching differences of more than 230 L p^−1^d^−1^ in the case of beef or 108 L p^−1^d^−1^ in milk and yogurt cases. The only food groups where the normal adiposity group presented a trend of higher WF were eggs (*p* = 0.155), vegetables (*p* = 0.543), sugars with fat (*p* = 0.075), oils with proteins (*p* = 0.394), and without proteins (*p* = 0.972).

The logistic regression analysis reporting odds ratios showed that having excess visceral fat produced 4.77 times (*p* < 0.001) greater risk of the Mexican diet, exceeding the WF of average healthy diets (2,714 L p^−1^d^−1^). BMI, body fat percentage, waist circumference, and waist–hip ratio were not statistically significant (Table 7).

**Table 7:** Logistic regression analysis reporting odds ratios of water expenditure risk regarding average healthy diet water footprint (WF) and adiposity indicators.

					***p*-value: 0.008**
	**OR**	**Std. Err**.	***z***	***p*****-value**	**[95% CI]**
BMI (kg m^−2^)	1.11	0.46	0.25	0.805	0.49 – 2.48
BF (%)	0.64	0.28	−1.02	0.308	0.27 – 1.51
WC (cm)	0.87	0.40	−0.30	0.765	0.36 – 2.13
VF (kg)	4.77	2.18	3.42	** <0.001**	1.95 – 11.67
WHR	0.57	0.23	−1.41	0.158	0.26 – 1.24

*BMI, Body mass index; BF, body fat percentage; WC, waist circumference; VF, visceral fat; WHR, waist-hip ratio*.

*Statistical significance was considered at p ≤ 0.05 and is in bold*.

*A confidence interval at 95%*.

### Water Footprint by Adherence to Dietary Recommendations

Table 8 shows the identified risks of exceeding the dietary WF of the healthy diet defined in this study (Figure 1) for surpassing the maximum recommended portion of consumption presented in Table 1. A consumption that exceeds the maximum recommended portion of red meats, including beef, pork, lamb, and processed meats (≤71.42 g p^−1^d^−1^), represented a significant risk of 92.93 times (*p* < 0.001) greater of exceeding the healthy diet WF. Likewise, exceeding the consumption of other foods of animal origin, such as milk and yogurt and cheese, generates risks up to 13.33 times (*p* < 0.001) greater of surpassing the WF of the healthy diet. Also, it was found that exceeding the recommended portion of consumption of natural and industrialized fruit juices generated a risk up to 4.64 times greater of exceeding the healthy diet WFs (*p* = 0.041). On the contrary, complying with the recommended rations of fish (*p* < 0.001), fruits and vegetables (*p* = 0.005), and non-fat cereals (*p* = 0.011) generates a protective factor in the generation of WF equals to or less than that of the healthy diet identified in this article (Table 8).

**Table 8:** Logistic regression analysis reporting odds ratios of water expenditure risk regarding average healthy diet water footprint (WF) and adherence to Mexican dietary recommendations.

					***p* < 0.001**
	**OR**	**Std. Err**.	***Z***	***p*-value**	**[95% CI]**
Milk and yogurt	13.33	8.08	4.27	** <0.001**	4.06 – 43.74
Cheeses	3.31	1.99	2.00	**0.046**	1.02 – 10.73
Poultry and eggs	1.99	0.89	1.54	0.124	0.83 – 4.77
Red meat	92.93	81.41	5.17	** <0.001**	16.69 – 517.47
Fish	0.23	0.10	−3.28	** <0.001**	0.09 – 0.55
Fruits and vegetables	0.21	0.12	−2.80	**0.005**	0.07 – 0.63
Legumes	0.47	0.21	−1.70	0.089	0.19 – 1.12
Cereals without fat	0.33	0.14	−2.53	**0.011**	0.14 – 0.78
CFS, FS, and NFS	0.59	0.36	−0.86	0.387	0.18 – 1.94
Nuts	0.65	0.29	−0.98	0.329	0.28 – 1.54
Fruit juice^a^	4.64	3.49	2.04	**0.041**	1.06 – 20.25
Soft drinks	1.13	0.48	0.28	0.776	0.49 – 2.61
Alcoholic drinks	0.73	0.35	−0.66	0.509	0.29 – 1.85

*CFS, Cereals with fat and/or sugars; FS, Fatty sugars; NFS, Not fatty sugars*.

a *Includes natural and industrialized fruit juice*.

*Statistical significance was considered at p ≤ 0.05 and is in bold*.

*A confidence interval at 95%*.

## Discussion

The environmental impact generated by different types of diets has shown that adherence to healthy diets can help reduce damage to the environment (20, 23). In this study, it was found that the Mexican diet generates a total water expenditure of 6,055.89 ± 2,719.24 L p^−1^d^−1^ and a WF from green and blue components of 5,487.38 ± 2,483.70 L p^−1^d^−1^. This last WF is comparable with the healthy diet identified in this study and exceeds it up to 2,773.38 L p^−1^d^−1^. The main differences between the healthy diets included in this study and the Mexican diet lie in the consumption of food of animal origin. In this study, it was identified that 23.38% of the food eaten comes from animal sources. While vegan diets do not include this kind of food, and vegetarians do not exceed 6% of animal foods in their diets (19). The Mediterranean diet, which is less drastic compared with the Mexican one with respect to meat and dairy consumption, reaches more than 10% of the food of animal origin, according to what was indicated by Blas et al. (18). So far, the only international diet that has exceeded the WF identified in this study is the one reported by Blas et al. (18) for the American diet but calculated in Spain, which exceeds the Mexican diet WF values by 724.11 L p^−1^d^−1^, considering green, blue, and grey WF, which correspond to a total WF of 6,780 L p^−1^d^−1^. In the context of Mexico, only one previous study has reported the WF of the Mexican diet, and the reported value is considerably higher than the one found here (8,334 L p^−1^d^−1^). However, the difference could lay in the method used, since 24-h recall was used, and we based on a FCFQ (16). Therefore, the value reported by Lares-Michel et al. (16) can be referred to as the “Mexican diet WF” and the found here as the Mexican dietary pattern WF.

The benefits of eating healthy for the environment are well-established (23, 24), but the advantages of maintaining adequate levels of body fat for the care of natural resources have been less studied, although, in recent years, this has attracted attention (6, 8, 66). The emission of greenhouse gases has been the ecological aspect most studied with respect to the environmental impact of adiposity on people (7, 67). Edwards and Roberts (68) pointed out that people with higher adiposity rates generate higher greenhouse gas emission, which is directly linked to the obesogenic environments that provide food with high environmental impact (8, 66). In addition, greater impacts on the ecological and carbon footprint have been reported, in contrast to people with normal levels of adiposity (8, 10, 66, 69).

However, the relationship of overweight and obesity with water use and WF, in particular, has been less studied, despite the significant water crisis that exists in many parts of the world (70), including Mexico, where the national availability of piped water per capita has reportedly been reduced since the 1970's, from 11,000 m^3^ annual per capita to 4,600 m^3^, and this figure is expected to decrease to 3,500 m^3^ per person by the year 2030 (71). Our results showed that having abdominal obesity by visceral fat generates a risk up to 4.77 times greater of exceeding the healthy diet WF. Therefore, as Abbade (66) points out, following a healthy diet can help promote public health by decreasing adiposity levels and attenuating the impact on the environment. However, if no changes are made in the diet, water availability in Mexico will be in serious trouble, since the WF of the Mexican diet had a standard deviation of 2,719.24 L p^−1^d^−1^. This means that some individuals of the sample generate a WF of more than 18,000 L p^−1^d^−1^ due to excessive meat consumption and hypercaloric diets.

To our knowledge, the present work represents the first empirical study available at an international level to evaluate the relationship that the adiposity of a sample has, in a real-life context, with the WF. That is, performing a direct evaluation of the diet of a group of people (25). Although Serafini and Toti (38) began to use WF as part of the metabolic food waste indicator, which evaluates the unsustainability of obesity, the direct relationship that adiposity, consumption of particular foods, and caloric intake of people in real life have with the WF has not yet been reported. In Mexico, there are no available studies linking overweight and obesity with water expenditure, despite this country being the most obese in the world (27).

According to our results, the population with excess adiposity generates a statistically significant higher WF than the population with normal adiposity (*p* < 0.05), which represents daily extra expenses of 726.86 L p^−1^d^−1^. This difference lied in the higher consumption of foods and calories, and, especially, in the consumption of food of animal origin such as cheese and lamb, which coincides with Serafini and Toti (38). Besides, it is important to note that although not statistically significant differences were found in the consumption of beef, the intake of this food group was higher in the excess adiposity group, which generated a WF 230 L p^−1^d^−1^ higher than the one found in the normal adiposity group. Also, statistically differences in soft drinks consumption and WF were found, but this intake represented only 1.08% of total WF. No available data were found to contrast these findings, but, since it is a kind of food whose principal ingredients are sweeteners that are of vegetable origin, its low WF is expected (72).

Besides the relationship identified between WF and excess adiposity, it is important to consider the high impact found in energy intake, especially in calories from food of animal origin. This has been reported in regard to ecological and carbon footprints (66), but the exact same analysis has not been made regarding WF. In contrast with available data, energy intake from the Mexican population proved to have a higher WF than other indicators. But further analysis, including other environmental indexes, is needed to provide a full picture of the Mexican-diet environmental impact.

In addition to calories, the type of food and the amounts ingested have high impacts on WF. Excessive food consumption, in particular, exceeding the recommended amounts of food for Mexico, represents the main problem for water expenditure. Red meats have been designated as the main foods that produces environmental impact (23, 38, 45). This coincides with our results, since beef consumption generated the greatest contribution to total and green WF, reaching up to 1,597 L p^−1^d^−1^ in the case of people with excess adiposity. The average population of this study consumes 96.18 ± 78.32 g p^−1^d^−1^ of red meat, while people with normal fat levels consume 89.05 ± 66.80 g p^−1^d^−1^, and those with excess adiposity with respect to BMI consume an average of 102.79 ± 87.31 g p^−1^d^−1^. This means that people with excess fat consume 143.92% of the recommendation. However, it is important to note that people with normal adiposity also exceed the suggested amount, consuming 124.68%. This study shows that this type of behavior increases the risk of exceeding a healthy-diet WF by 92.93 times. Red meat consumption has been reiterated as the principal problem in environmental impacts of diets, as well as dairy consumption (73). Indeed, our results agree, because besides exceeding red meat consumption, the average population consumes 107.92% of the suggested amount of consumption of milk and yogurt, and the excess adiposity population consumes 115.84%. This consumption generated the second-largest WF. Besides, cheese WF was significantly higher in the excess adiposity group. According to Macdiarmid et al. (73), small reductions in meat and dairy consumption can reduce the environmental impact of diets, but this was assessed specifically on greenhouse gas emission, so further studies related to WF are needed. Also, it is important to consider that the recommended amount of consumption of red meat in Mexico (Table 1) is substantially different from the “planetary health diet” recently recommended (74). That diet, which was proposed by The EAT-Lancet commission, is a scientifically optimized diet for both nutrition and certain environmental indicators, which have been proved to have the potential of reducing up the dietary WF of diets. However, cultural, social and economic aspects remain unclear in those recommendations (75). Therefore, the Mexican government needs to rethink the dietary recommendations that are providing for their population and take into consideration evidence on dietary WF reductions but considering cultural, social and economic aspects. In this sense, meat consumption reduction is a challenge, since its consumption can be linked with cultural aspects and even with social status (76).

Another interesting finding was identified in some drinks. The recommendation in regard to sugary drinks, especially soft drinks and fruit juices, is to avoid ingesting or, at most, consuming them in a maximum amount of 34.28 and 125 ml p^−1^d^−1^, respectively (65). In the case of fruit juices, the consumption of the population did not exceed the recommended portion since their intake represented 53.21% of the recommendation. However, exceeding it resulted in a risk of 4.64 times greater water expenditure regarding healthy diet WF. Regarding soft drinks, the population did exceed the recommended amount of consumption since their ingestion reaches 537.11% of these.

Besides the contention that excessive consumption of inadequate food causes a risk for water expenditure, it should be taken into account that the consumption of healthy foods below the recommended amounts also results in a failure to meet the protective factors identified in this study, especially in fish, fruits, vegetables, and cereals without fat consumption. These findings suggested that eating well is a means of saving water. However, in these cases, a paradoxical outcome was identified since fruits were the most consumed food group both in the general population and in adiposity groups. Also, in cereals without fat and fish and seafood, minimum rations of consumption were overpassed in both adiposity groups and the general population. This can be a warning about the way of communicating dietary recommendation since the population without enough nutritional education and criterion are likely to overpass consumption of food that, although are considered healthy, can lead to overweight and obesity if consumption exceeded the energy requirements of each individual (77). Therefore, the amounts of consumption as well as caloric intake are important findings in this study, which proved that not only types of foods matter, and eating right but always in adequate amounts can prevent the future development of obesity and, especially, overexploitation of water resources.

In this regard, overconsumption must be closely analyzed since, besides being the main cause of obesity (12), this could be an important environmental impact generator in food systems (78). The reference dietary guideline for Mexico (79) recommends an average caloric intake for the adult population of 2,188 Kcal p^−1^d^−1^. Our results found a caloric intake of 2,415 Kcal p^−1^d^−1^ for the general population, 2,294 Kcal p^−1^d^−1^ in the population with normal adiposity, and 2,528 Kcal p^−1^d^−1^ in people with excess adiposity, which was found as statistically different regarding the normal adiposity group (*p* = 0.008). This represented overconsumption of 16% from recommended caloric intake and an extra water expenditure that was 13% higher than the population with normal adiposity. However, it is important to mention that even people with normal adiposity consumed higher amounts of calories in comparison with the suggested amount (79). This is an explanation of the Mexican dietary pattern WF being among the highest worldwide in this study and in Lares-Michel et al. (16). Therefore, besides promoting healthy eating regarding food types, quantities ingested are very important factors in WF generation.

Also, is important to bring to attention that even healthy foods, such as vegetables and fruits, although not generating the highest total WF impacts, are demanding a lot of water use, especially blue WF (25). So, based on this, some authors that have started to evaluate the impact of nutritional interventions on environmental indicators have found that improvements in the diet do not necessarily reduce environmental impacts (80). This reason is why achieving sustainable and realistic diets is a big challenge, because, although our findings support the notion that healthy diets can be sustainable [at least in the nutrition, health, and environmental (WF) domains], this is not always the case (9, 73), and, actually, more studies in other populations are needed in order to explore different dietary patterns and their environmental impacts and water use.

With respect to the healthy diets that were included in this study as a reference point for water expenditure risk analysis, it is important to analyze the viability of the Mexican population to adhere to one of those to achieve diets with less WF. The consumption of foods of animal origin has been referred to as an important element in the Mexican diet (81). Therefore, the adherence of the Mexican population to a vegan diet (19) is not realistic. Vegetarian diets resulted less drastic than vegan diets since the analyzed vegetarian diets included dairy and eggs (19, 20), and pesco-vegetarian, besides those, included fish and seafood (24, 45). However, it is important to note that vegetarian diets are not always healthy; therefore, their healthy versions could be a better strategy for both, improving health and water use, since this type of diet regulates in a better way amounts of consumption, especially of fats and sugars, that, although could have low WF, are not healthy in high amounts (24). Although adherence to vegetarian and healthy vegetarian could be a good strategy for the Mexican population to decrease their dietary WF, the analyzed healthy diets that included meat in adequate amounts could be a more realistic option for a population that is used to consume this type of foods.

The healthy diets included (20, 21, 45) that referred to a general healthy diet, indicates the suggested amount of consumption of cereals, rice, potatoes, and pulses (bread 70–245 g p^−1^d^−1^ and other 50–250 g p^−1^d^−1^), sugar (max 50 g p^−1^d^−1^), vegetables (50–200 g p^−1^d^−1^), fruits (150–200 g p^−1^d^−1^), meat (including offals), fish and seafood, eggs, nuts, and oil crops (50–125 g p^−1^d^−1^), animal fats and crop oils (10–30 g p^−1^d^−1^ and 15 g p^−1^d^−1^ for cooking), milk and milk products (300–650 ml p^−1^d^−1^ and 10–30 g p^−1^d^−1^ of cheese), and alcoholic beverages [max 20 ml p^−1^d^−1^ of pure alcohol for men (two standard drinks) and max 10 ml p^−1^d^−1^ of pure alcohol for women (one standard drink)] (45). These amounts of consumption are similar to Mexican dietary recommendations (Table 1) in regard to cereals and legumes. However, the recommendations of the healthy diets establish a maximum amount (20, 21, 45), while Mexican recommendations only suggest consuming 200 g p^−1^d^−1^ (63). This could be an important element in the development of overweight and obesity. In the case of milk and milk products, the recommendation is higher in the healthy diet, in contrast to the Mexican recommendations. However, something to point out is that the maximum amount of consumption of foods of animal sources (nuts are included in that category in healthy diet recommendations) in healthy diets is 125 g p^−1^d^−1^, and, in the Mexican recommendations, is 160.66 g p^−1^d^−1^. This is an important consideration for Mexican dietary guidelines to rethink the recommendations given to promote more sustainable diets. Nevertheless, it is important to point out that water savings through dietary changes are not as simple as what could look alike. That is, regional production can have important variations that can be constantly moving according to climate conditions and, it is also necessary to consider the strategies that will have to be applied to supply an increasing demand of certain products, such as fruits and vegetables, and the implications of imports to supplement the local production. This definitely shows the complexity of food systems, especially when they are related to obesity and healthy eating (15, 82).

One of the diet scenarios that could fit the Mexican population is the Mediterranean diet (18, 44) and the healthy meat included, which is also based on the Mediterranean diet (24). The Mediterranean diet has been reported as one of the most healthy and sustainable diets worldwide, and, also, this diet has been used by the Mexican government to develop some of the current dietary recommendations (64, 83). This diet has certain similarities to the Mexican one since it includes red meat (in moderate amounts), milk, cheese, fish, fruits, and vegetables. However, some particularities of the Mexican diet, as corn as the base of its diet as well as beans, are not similar, since the base cereal of the Mediterranean diet is wheat (84). Therefore, for the Mexican dietary context, it is necessary to update the current dietary guidelines or develop new ones that establish the healthiness and sustainability of the Mexican diet.

Despite the idea of healthy eating as a means of reducing the environmental impact of diets has been widely disseminated (20, 85), these effects can vary greatly in the regard of environmental impact and water use specifically, and, aside from what has been referred to, healthy diets could generate a higher impact and WF than other types of diets (86). Although following dietary recommendations are one of the most common ways of promoting healthy and sustainable eating, for some countries, this does not support environmental impact reduction. As example of other environmental indicators, Grasso et al. (80) found that attaching to the Mediterranean diet can have an unfavorable increase in fossil energy use and can generate the same environmental impact as current diets in regard to greenhouse gas emissions. In the context of WF, United States is an example that healthy diets do not always have a low environmental impact, since it has been referred that adhering to national dietary guidelines does not represent less environmental impact than current diets (87). In this sense, Birney et al. (88) found that shifting diets from current consumption to the USDA dietary guideline recommendations would result in an increase in blue water use by 15%. Also, it could increase the environmental impact of other environmental indicators, for example, an increase of 34% in energy use, 7% in greenhouse gas emissions of food production, and 34% in fertilizer use. Although these findings, is important to point out that blue WF reported in that study was 91 L p^−1^d^−1^ lower than the one found in our study for the general population and 114 L p^−1^d^−1^ lower than the one found for the excess adiposity group. Therefore, adopting dietary recommendations for the Mexican population could represent, although little, decrease in water use. The results of Birney et al. (88) are consistent with several studies (23, 87, 89, 90); however, implementing other changes, such as caloric intake reduction, reducing more animal foods, and reducing food waste, could significantly decrease WF in comparison with only adopting a healthy diet (89).

This study represents one of the first efforts to elucidate the relationship between obesity and the environmental impact of diets, specifically on water use. To our knowledge, this is the first investigation to compare the WF of the diet of people with different degrees of adiposity in a real-life context. However, our work has some limitations. First, it is important to mention that, although we evaluated a representative sample of Jalisco, which is an important state of Mexico, this study does not have national representativeness, so further studies are needed in bigger samples. Second, although the FCFQ used is specific for the zone of the study, it is outdated since it does not include a large amount of traditional and western foods that are consumed by the Mexican population. Therefore, new tools development is needed to provide a deeper analysis of the Mexican diet and its cultural diversity, as well as its degree of westernization and, of course, its impact on WF. Third, BMI as an adiposity indicator has been pointed out as inadequate since it does not directly measure fat (91). For this reason, this parameter was associated with adiposity indicators. However, future analysis, using other adiposity indicators for sampling, is needed to confirm the impact of adiposity on WF. Fourth, WF used data is specific to Mexico and Jalisco, and food imports and exports were not considered, as these have been the most used WF calculation method worldwide (25). Nevertheless, future studies are required to identify if the WF of the Mexican diet actually comes from the country or from other parts of the world. Fifthly, although WF is a valuable index that is widely used for assessing dietary environmental impacts, there are other important indicators that were not considered in this study. These include greenhouse gas emissions, land use, nitrogen and phosphorus use, and biodiversity loss, among others (74). Therefore, more studies relating obesity and dietary intake with other environmental indicators besides WF are needed.

Finally, and although we did not address a whole dietary environmental assessment, our results support other studies that have linked diets with environmental impacts, including not only WF but other environmental indicators such as the aforementioned. In this sense, unhealthy and inadequate eating besides being driven to the obesity pandemic, is contributing negatively to water use (85, 92), climate change (10, 85, 92), land use (85, 92), and nitrogen and phosphorus use (92). And these problems are not only affecting specific zones, such as Mexico, but the whole world. Therefore, it is urgent that public policies in Mexico and around the world begin to focus on providing dietary recommendations that allow for the care of water resources, putting into practice the recommendations that this study identified as protective factors in water and regulating the consumption of those foods that represented risks to the WF. Although it has been suggested that a “fat tax” could levy on unhealthy food choices and could be a possible solution to promote healthy lifestyles (7), we state that it is important not to stigmatize the obese population; therefore behavioral change interventions and educational programs in regard to sustainable nutrition are the key elements to modify antienvironmental dietary behaviors. In this way, we consider future nutritional interventions, in addition to promoting healthy eating to reduce obesity, could help to resolve these issues, along with the resolution of one of the main problems in Mexico and throughout the world: water scarcity. This way and in agreement with emerging studies, we could be contributing to achieve both the United Nations Sustainable Development Goal (SDG) 2 on food security and SDG 6 on water security in a water-energy-food-ecosystem nexus, especially regarding the reduction of global water scarcity (75).

## Conclusion

This study shows that the adherence to the dietary recommendations of Mexico regarding the consumption of fruits and vegetables, fish and seafood, and non-fat cereals, such as corn tortillas, whole-grain bread, and rice, generated a protective factor in WF that is equal to or less than the healthy diet found in this study, which, considering only green and blue WF, was 2,714 L p^−1^d^−1^. Consumption exceeding the suggested amounts of red meat, milk and yogurt, cheeses, and natural and industrialized fruit juices generated a risk up to 92.93 times greater of exceeding WF of the healthy diet. Likewise, it was found that the more hypercaloric the diet, the greater the WF, and, especially, calories coming from food of animal sources generated higher impacts on WF. In addition, one of the most important findings is that having excess adiposity by visceral fat represented a risk up to 4.77 times greater of generating WFs above the healthy-diet water expenditure. Besides, the diet WF of people with excess adiposity was statistically higher than dietary WF of the normal adiposity population, which generated additional water expenses of up to 726.86 L p^−1^d^−1^. For this, it is concluded that excess adiposity, besides being related to public health and economic implications, also has important environmental impacts, especially on WF. However, further studies are needed to provide a full picture in regard to other environmental indicators and other and bigger samples that allow assessing the impact that the obesity epidemic has on the environment. As a sensitive topic, overweight and obesity need to be targeted by multidisciplinary teams, whose focus is eating behavior and nutrition. But, currently, health professionals are required to know more about the environmental impact that food production and diets have and apply that knowledge to fight both, nutritional and environmental problems.

## Data Availability Statement

The datasets generated for this study are available on request to the corresponding author.

## Ethics Statement

This study was approved by the Ethics Committee of the University of Guadalajara CEICUC (registration number CEICUC-PGE-004). Also, the principles of the Helsinki declaration were followed, and all the participants were adults who signed an informed consent before being included in the study.

## Author Contributions

ML-M and FEH conceived the idea and design of the study. ML-M led data collection, analysis and interpretation and wrote the article. FEH and VGAC provided help on data acquisition, interpretation, and critical review of the first draft. VGAC, PC, RMMN, and CLC aided in the design of the study, interpretation of data, and critical revision of the manuscript. All authors read and approved the submitted version of the manuscript.

## Conflict of Interest

The authors declare that the research was conducted in the absence of any commercial or financial relationships that could be construed as a potential conflict of interest.

## Supplementary Material

Supplementary Materials show complementary information for the article.
