# Supplementary material for: Eat Well to Fight Obesity… and Save Water: The Water Footprint of Different Diets and Caloric Intake and Its Relationship With Adiposity
Source: Front Nutr. 2021 Jul 1;8:694775. doi: 10.3389/fnut.2021.694775 (PMC8281344; doi:10.3389/fnut.2021.694775)
Supplement: Supplementary file 1 [file Data_Sheet_1.docx]

Supplementary Material

**Supplementary material 1. Calculation of a representative sample from Jalisco**

**Population**

The universe of this study corresponds to the adult population of the state of Jalisco, between 18 and 74 years of age, which corresponds to 5,016,194 million people, according to the Institute of Statistical and Geographic Information of Jalisco (1).

**Sample size**

The following probabilistic formula was applied (2,3), with a confidence level of 95% and a margin of error of 5%, for a finite and known population, greater than 10,000 inhabitants:

$$n=\frac{Z^{2}.p.\left( 1-p \right)}{e^{2}}$$

Where:

- n: sample size
- z: value corresponding to the gauss distribution z = 0.05 = 1.96
- p: expected prevalence of the parameter to be evaluated, if unknown (p = 0.5)
- e = 0.05 (5 % margin of error)

With which a study sample of 384 people was obtained.

**Supplementary material 2. Foods included in the Food Consumption Frequency Questionnaire**

| Food group | Food |
| --- | --- |
| Milk and yogurt | 1. Whole milk |
|  | 2. Semi-skim milk |
|  | 3. Skim milk |
|  | 4. Packaged milk shake |
|  | 5. Whole yogurt (sweetened with fruit) |
|  | 6. Low-fat yogurt |
|  | 7. Petit suisse |
|  | 8. Fermented milk drink |
|  | 9. Evaporated milk |
| Cheeses | 10. Curd |
|  | 11. Cottage cheese |
|  | 12. White or fresh cheese (goat, *adobera*, panela) |
|  | 13. Other cheeses: cured, semi-cured (*manchego*, gouda, *oaxaca*, mozzarella) |
| Eggs | 14. Chicken eggs |
| Chicken | 15. Chicken with skin |
|  | 16. Chicken without skin |
|  | 17. Turkey with skin |
|  | 18. Turkey without skin |
|  | 19. Liver or organ meats of chicken (menudo, brains, heart, gizzards) |
| Beef | 20. Beef |
|  | 21. Hamburger meat |
|  | 22. Meatballs |
|  | 23. Liver or organ meats of beef (menudo, brains, heart, gizzards) |
| Pork | 24. Pork meat |
|  | 25. Liver or organ meats of pork (menudo, brains, heart, gizzards) |
| Lamb | 26. Sheep meat |
| Process meats | 27. Cooked ham (pork, turkey, etc.) |
|  | 28. Turkey or pork sausage |
|  | 29. Processed meats (salami, *chorizo, longaniza*, stuffed, moronga, mortadella) |
|  | 30. Bacon |
|  | 31. Pork rinds |
| Fish and seafood | 32. White fish (grouper, sole, bream, crappie) |
|  | 33. Blue fish (sardines, tuna, salmon, red snapper, catfish) |
|  | 34. Salty fish (cod, *charales*) |
|  | 35. Canned fish and shellfish in water (sardines, anchovies, tuna, salmon) |
|  | 36. Canned fish and shellfish in oil (sardines, anchovies, tuna, salmon) |
|  | 37. Prepared fish and shellfish (with tomatoes sauce, salad, others) |
|  | 38. Crustaceans: shrimp, prawns |
|  | 39. Octopus or squid |
|  | 40. Oysters, oysters, clams, mussels |
| Vegetables | 41. Swiss chard, spinach or purslane |
|  | 42. Cabbage, cauliflower, broccoli |
|  | 43. Lettuce, endives |
|  | 44. Red tomato raw or in sauce |
|  | 45. Carrot |
|  | 46. Zucchini |
|  | 47. Pumpkin |
|  | 48. Green beans or peas |
|  | 49. Cucumber |
|  | 50. Celery, artichoke, leek, thistle |
|  | 51. Beetroot |
|  | 52. Eggplant |
|  | 53. Asparagus |
|  | 54. Onion |

**Supplementary material 2. Continuation: Foods included in the Food Consumption Frequency Questionnaire**

| Food group | Food |
| --- | --- |
| Vegetables | 55. Green tomato, raw or in sauce |
|  | 56. Mushrooms and champignons |
|  | 57. *Chayote* |
|  | 58. Jicama |
|  | 59. Cooked, raw or roasted *nopales* (cactus) |
|  | 60. Bell pepper |
|  | 61. Poblano Chile |
|  | 62. Hot peppers (jalapeño, serrano, habanero) |
|  | 63. Garlic |
|  | 64. Parsley, thyme, bay leaf, oregano, coriander, spearmint, mint, basil |
|  | 65. Pumpkin flower |
| Fruits | 66. Orange (not in juice) |
|  | 67. Grapefruit (not in juice) |
|  | 68. Tangerines (not in juice) |
|  | 69. Lemon |
|  | 70. Lime (not in juice) |
|  | 71. Banana (not fried) |
|  | 72. Apple |
|  | 73. Pear |
|  | 74. Strawberries |
|  | 75. Plums |
|  | 76. Cherries |
|  | 77. Peach |
|  | 78. Nectarine, apricot |
|  | 79. Watermelon |
|  | 80. Cantaloupe |
|  | 81. Papaya |
|  | 82. Kiwi |
|  | 83. Grapes |
|  | 84. Mango |
|  | 85. Guava |
|  | 86. Tuna |
|  | 87. Pineapple |
|  | 88. Tamarind |
|  | 89. Canned fruit in syrup |
|  | 90. Dates, dried figs, prunes, raisins |
| Oils with protein or protein oils | 91. Almonds |
|  | 92. Peanuts (salty, with chillie, garapiñados) |
|  | 93. Nuts |
|  | 94. Pistachios, hazelnuts, pine nuts |
|  | 95. Sunflower or pumpkin seeds |
| Legumes | 96. Cooked beans |
|  | 97. Haricot (pin, white or black) |
|  | 98. Cooked lentils |
|  | 99. Cooked chickpeas |
|  | 100. Broad beans |
| Cereals without fat or non-fat cereals | 101. Corn tortilla |
|  | 102. Corn dough |
|  | 103. Corn cob |
|  | 104. Roasted or cooked potato |
|  | 105. Flour tortilla |
|  | 106. Bolillo, *birote* or *telera* |
|  | 107. White bread |
|  | 108. Whole wheat bread |
|  | 109. Breakfast cereals |

**Supplementary material 2. Continuation: Foods included in the Food Consumption Frequency Questionnaire**

| Food group | Food |
| --- | --- |
| Cereals without fat or non-fat cereals | 110. Whole grains cereals: muesli, all-bran |
|  | 111. Pasta (noodles, macaroni, spaghetti) |
|  | 112. Oatmeal (flaked, cooked in water) |
|  | 113. Cooked white rice |
|  | 114. Marias cookies |
|  | 115. Whole grain or fiber cookies |
|  | 116. Bread for hamburger |
|  | 117. Bread for hot dogs |
| Cereals with fat or fatty cereals | 118. Fried corn toast |
|  | 119. Chocolate cookies |
|  | 120. Granola with nuts |
|  | 121. Homemade breads |
|  | 122. Hot cake |
|  | 123. Commercial industrialized bread (cinnamon rolls, *chocorroles*, rolls, package donuts, muffins) |
|  | 124. Sweet bread (*conchas, orejas, moños, polvorón, semas*) |
|  | 125. Cake |
|  | 126. Industrialized donuts |
|  | 127. Shortbreads |
|  | 128. Sugary churros |
|  | 129. Pizza |
|  | 130. Packaged chips |
|  | 131. Packaged corn snack |
|  | 132. French fries or home fries |
|  | 133. Wheat *churritos* |
|  | 134. Popcorn (homemade, microwave, cinema) |
|  | 135. Envelope soups and creams |
| Oils without protein | 136. Olive oil |
|  | 137. Extra virgin olive oil |
|  | 138. Corn oil |
|  | 139. Sunflower oil |
|  | 140. Soy oil |
|  | 141. Mix of the above |
|  | 142. Canola oil |
|  | 143. Safflower oil |
|  | 144. Olives |
|  | 145. Avocado |
|  | 146. Mayonnaise, dressing, mustard or vinaigrette |
|  | 147. Margarine |
|  | 148. Butter |
|  | 149. Lard |
|  | 150. Vegetable shortening |
|  | 151. Cream cheese |
|  | 152. Cream, nata or *jocoque* |
| Sugars without fat or not fatty sugars | 153. Sugar |
|  | 154. Honey |
|  | 155. *Cajeta* |
|  | 156. Condensed milk |
|  | 157. Piloncillo (brown sugar) |
|  | 158. Jams |
|  | 159. Gelatin in water |
|  | 160. Packaged candy (gummies, lollipops, commercial candy) |
|  | 161. Ketchup sauce or canned tomato puree |
|  | 162. Bottled hot sauce |

**Supplementary material 2. Continuation: Foods included in the Food Consumption Frequency Questionnaire**

| Food group | Food |
| --- | --- |
| Sugars with fat or fatty sugars | 163. Chocolates |
|  | 164. Cocoa powder for chocolate milk |
|  | 165. Ice cream or ice cream popsicle |
|  | 166. Mexican candy (*Ate, cocada,* milk candy, Tamarindo candy) |
|  | 167. Custard, flan, *jericalla* |
| Soft drinks | 168. Carbonated drinks with sugar: cola, flavored soda, lemonades |
|  | 169. Low calorie carbonated drinks, light drinks: light sodas, mineral water, etc. |
| Fruit juice | 170. Natural fruit juice of orange |
|  | 171. Natural fruit juice of other fruits (grapefruit, tangerine) |
|  | 172. Bottled or canned industrialized fruit juices |
| Coffee and tea | 173. Decaffeinated express coffee |
|  | 174. Espresso coffee |
|  | 175. Soluble coffee, decaffeinated coffee |
|  | 176. Tea |
| Alcoholic drinks | 177. Rose wine |
|  | 178. Young red wine, of the year |
|  | 179. Old red wine |
|  | 180. White wine |
|  | 181. Beer |
|  | 182. Liquors: anise, amaranth, coffee, etc. |
|  | 183. Distillate: whiskey, vodka, gin, brandy, tequila, mezcal, rum, brandy |
| Natural water | 184. Natural water |

**Supplementary material 3. Compilation of the water footprint of different healthy diets worldwide**

Note: Mediterranean city (MC); * green and blue WF only.

| Diet type | Place | WF (L p^-1^ d^-1^) | Method | | WF type | | Source |
| --- | --- | --- | --- | --- | --- | --- | --- |
|  |  |  | WFA | LCA | Green and blue | Green, blue and grey |  |
| Healthy  (general) | Amsterdam, Netherlands | 2,220 |  |  |  |  | (4) |
|  | Austria | 3,655 |  |  |  |  | (5) |
|  | Dordrecht, Netherlands | 2,214 |  |  |  |  | (4) |
|  | Eindhoven, Netherlands | 2,204 |  |  |  |  | (4) |
|  | Maastricht, Netherlands | 2,222 |  |  |  |  | (4) |
|  | Nieuwegein, Netherlands | 2,199 |  |  |  |  | (4) |
|  | Rotterdam, Netherlands | 2,215 |  |  |  |  | (4) |
|  | European union | 3,291 |  |  |  |  | (6) |
|  | European union* | 2,979 |  |  |  |  | (6) |
|  | Venlo, Netherlands | 2,205 |  |  |  |  | (4) |
|  | Average (EU excluded) | 2,457 |  |  |  |  |  |
| Healthy with  meat | Ankara, Turkey (MC) | 3,090 |  |  |  |  | (7) |
|  | Ankara, Turkey | 4,115 |  |  |  |  | (7) |
|  | Atenas, Greece (MC) | 3,170 |  |  |  |  | (7) |
|  | Bologna, Italy (MC) | 2,875 |  |  |  |  | (7) |
|  | Dubrovnik, Croatia (MC) | 3,654 |  |  |  |  | (7) |
|  | Genova, Italia | 2,882 |  |  |  |  | (7) |
|  | Istanbul, Turkey (MC) | 3,090 |  |  |  |  | (7) |
|  | Istanbul, Turkey | 4,115 |  |  |  |  | (7) |
|  | Jerusalem, Israel (MC) | 3,285 |  |  |  |  | (7) |
|  | Ljubljana, Slovenia (MC) | 2,459 |  |  |  |  | (7) |
|  | Lyon, France (MC) | 2,363 |  |  |  |  | (7) |
|  | Manresa, Spain (MC) | 3579 |  |  |  |  | (7) |
|  | Pisa, Italy (MC) | 2796 |  |  |  |  | (7) |
|  | Reggio (nell')Emilia, Italy (MC) | 2872 |  |  |  |  | (7) |
|  | Zaragoza, Spain (MC) | 3,580 |  |  |  |  | (7) |
|  | Average | 3,195 |  |  |  |  |  |
| Mediterranean | Spain | 5,276 |  |  |  |  | (8) |
|  | Spain (green and blue only) | 4,821 |  |  |  |  | (8) |
|  | United States of America | 4,003 |  |  |  |  | (8) |
|  | United States of America (green and blue only) | 3,213 |  |  |  |  | (8) |
|  | Italy | 1,968.71 |  |  |  |  | (9) |
|  | Average | 3,749 |  |  |  |  |  |
|  | Average (Italy excluded) | 4,640 |  |  |  |  |  |
|  | Average (green and blue only) | 4,017 |  |  |  |  |  |

**Supplementary material 3. Continuation: Compilation of the water footprint of different healthy diets worldwide**

| Diet type | Place | WF (L p^-1^ d^-1^) | Method | | WF type | | Source |
| --- | --- | --- | --- | --- | --- | --- | --- |
|  |  |  | WFA | LCA | Green and blue | Green, blue and grey |  |
| Vegetarian  (general) | Amsterdam, Netherlands | 1,883 |  |  |  |  | (4) |
|  | Austria | 2,293 |  |  |  |  | (5) |
|  | Dordrecht, Netherlands | 1,876 |  |  |  |  | (4) |
|  | Eindhoven, Netherlands | 1,863 |  |  |  |  | (4) |
|  | Italy | 2,304 |  |  |  |  | (10) |
|  | Maastricht, Netherlands | 1,873 |  |  |  |  | (4) |
|  | Nieuwegein, Netherlands | 1,860 |  |  |  |  | (4) |
|  | Rotterdam, Netherlands | 1,877 |  |  |  |  | (4) |
|  | European Union (EU) | 2,655 |  |  |  |  | (6) |
|  | European Union (EU)* | 2,394 |  |  |  |  | (6) |
|  | Venlo, Netherlands | 1,864 |  |  |  |  | (4) |
|  | Average (Italy and EU excluded) | 1,976 |  |  |  |  |  |
| Healthy vegetarian | Ankara, Turkey (MC) | 2,510 |  |  |  |  | (7) |
|  | Ankara, Turkey | 2,552 |  |  |  |  | (7) |
|  | Atenas, Greece (MC) | 2,752 |  |  |  |  | (7) |
|  | Bolonia, Italia (MC) | 2,518 |  |  |  |  | (7) |
|  | Dubrovnik, Croacia (MC) | 3,194 |  |  |  |  | (7) |
|  | Génova, Italia (MC) | 2,524 |  |  |  |  | (7) |
|  | Estambul, Turkey (MC) | 2,510 |  |  |  |  | (7) |
|  | Estambul, Turkey | 2,557 |  |  |  |  | (7) |
|  | Jerusalén, Israel MC) | 2,708 |  |  |  |  | (7) |
|  | Liubliana, Eslovenia (MC) | 2,211 |  |  |  |  | (7) |
|  | Lyon, Francia (MC) | 2,076 |  |  |  |  | (7) |
|  | Manresa, Spain (MC) | 3,183 |  |  |  |  | (7) |
|  | Pisa, Italia (MC) | 2,438 |  |  |  |  | (7) |
|  | Reggio Emilia, Italia (MC) | 2,515 |  |  |  |  | (7) |
|  | European Union (EU) | 2,973 |  |  |  |  | (6) |
|  | European Union (EU)* | 2,686 |  |  |  |  | (6) |
|  | Zaragoza, Spain (MC) | 3,184 |  |  |  |  | (7) |
|  | Average (EU excluded) | 2,632 |  |  |  |  |  |
| Pesco-vegetarian | Amsterdam, Netherlands | 1,999 |  |  |  |  | (4) |
|  | Dordrecht, Netherlands | 1,992 |  |  |  |  | (4) |
|  | Eindhoven, Netherlands | 1,979 |  |  |  |  | (4) |
|  | Maastricht, Netherlands | 1,992 |  |  |  |  | (4) |
|  | Nieuwegein, Netherlands | 1,976 |  |  |  |  | (4) |
|  | Rotterdam, Netherlands | 1,994 |  |  |  |  | (4) |
|  | Venlo, Netherlands | 1,981 |  |  |  |  | (4) |
|  | Average | 1,988 |  |  |  |  | (4) |

Note: Mediterranean city (MC); * green and blue WF only.

**Supplementary material 3. Continuation: Compilation of the water footprint of different diet types worldwide**

| Diet type | Place | WF (L p^-1^ d^-1^) | Method | | WF type | | Source |
| --- | --- | --- | --- | --- | --- | --- | --- |
|  |  |  | WFA | LCA | Green and blue | Green, blue and grey |  |
| Healthy pesco-vegetarian | Ankara, Turkey (MC) | 2,594 |  |  |  |  | (7) |
|  | Ankara, Turkey | 2,711 |  |  |  |  | (7) |
|  | Atenas, Greece | 2,853 |  |  |  |  | (7) |
|  | Bolonia, Italia (MC) | 2,604 |  |  |  |  | (7) |
|  | Dubrovnik, Croacia | 3,283 |  |  |  |  | (7) |
|  | Génova, Italia (MC) | 2,611 |  |  |  |  | (7) |
|  | Estambul, Turkey (MC) | 2,594 |  |  |  |  | (7) |
|  | Estambul, Turkey | 2,716 |  |  |  |  | (7) |
|  | Jerusalén, Israel | 2,805 |  |  |  |  | (7) |
|  | Liubliana, Eslovenia | 2,309 |  |  |  |  | (7) |
|  | Lyon, Francia | 2,163 |  |  |  |  | (7) |
|  | Manresa, Spain | 3,304 |  |  |  |  | (7) |
|  | Pisa, Italia | 2,526 |  |  |  |  | (7) |
|  | Reggio Emilia, Italia | 2,601 |  |  |  |  | (7) |
|  | Zaragoza, España | 3,306 |  |  |  |  | (7) |
|  | Average | 2,732 |  |  |  |  |  |

Note: Mediterranean city (MC).

**Supplementary material 4. Definition and characteristics of healthy diets**

| Diet type | Place | Definition/characteristics | Comments | Source |
| --- | --- | --- | --- | --- |
| Healthy (general) | Dutch cities | The one recommended by  the Dutch Food Based Dietary Guidelines (FBDG) | Specific amounts of recommend consumption of cereals, rice, potatoes, pulses, sugar, vegetables, fruit, meat (including offal’s), fish and seafood, eggs, nuts and oil crops, animal fats and crop oils, milk and milk products, stimulants, spices and alcoholic beverages are detail in supplementary materials of (4) | (4) |
|  | Austria | It is based upon the dietary recommendations  issued by the Deutsche Gesellschaft für  Ernährung or DGE – German nutrition society | Details are present in (5) | (5) |
|  | European Union | It is based upon the dietary  recommendations issued by the  Deutsche Gesellschaft für Ernährung (DGE) – German nutrition society | Details are present in (6) | (6) |
| Healthy with meat | Mediterranean cities | It is based on the Mediterranean  diet dietary recommendations. For the two Turkish cities Ankara and Istanbul of this study, the authors used additionally used Turkish Food-Based Dietary guidelines (FBDG) | Details are present in (7) | (7) |
| Mediterranean | Spain and United States | To characterize the composition and product quantities of the Mediterranean diet, two seasonal weeklong menus were defined (i.e., winter and summer) using the food guidelines elaborated by  the Mediterranean Diet Foundation | Details are present in supplementary material of (8) | (8) |
|  | Italy | The Mediterranean diet model is the result of a scientific consensus among experts who developed a new revised edition of the Mediterranean pyramid based on the model of Mediterranean diet | Details are present in (9) | (9) |
| Vegetarian (general) | Dutch cities | It is identical as the Pesco-vegetarian (based in Dutch Food Based Dietary Guidelines) but all fish is substituted with products from the pulses group (with the same kcal and protein values). | Details are specified in supplementary materials of (4) | (4) |

**Supplementary material 4. Continuation: Definition and characteristics of healthy diets**

| Diet type | Place | Definition/characteristics | Comments | Source |
| --- | --- | --- | --- | --- |
| Vegetarian (general) | Austria | Has the composition as the healthy diet (general), but all meat products are substituted by pulses and oil crops. Dairy products are still of animal origin (due to the economic and ecological importance of dairy  production on the grasslands and meadows of Austria) | Details are present in (5) | (5) |
|  | Italy | Dietary data was obtained from direct dietetic information of individuals following vegan and vegetarian diets. The Italian Mediterranean Index was used to evaluate the level of adherence to the Mediterranean dietary pattern, a measure of the participants’ diet healthiness. | Details are present in (10) | (10) |
|  | European Union | Same as the healthy diet, but all meat products are substituted by pulses and oil crops. Dairy products are still of animal origin | Details are present in (6) | (6) |
| Healthy vegetarian | Mediterranean cities | It is based on Bach-Faig et al. (2011a) and Ministry of Health of Turkey and HUNDD (2006) | Details are present in (7), including cites of Bach-Faig et al. (2011a) and Ministry of Health of Turkey and HUNDD (2006) | (7) |
|  | European Union | Diet between a healthy and vegetarian diet: half of the meat products is replaced by pulses and oil crops | Details are present in (6) | (6) |
| Pesco-vegetarian | Dutch cities | It is identical as the Healthy general (based in Dutch Food Based Dietary Guidelines) but all meat and offal’s are substituted with products from the product group pulses including oil crops (beans, peas, soybeans, etc.). Animal fats are substituted with crop oils. All these substitutions result in the same total kcal and protein values. | Details are specified in supplementary materials of (4) | (4) |
| Healthy pesco-vegetarian | Mediterranean cities | It is based on Bach-Faig et al. (2011a) and Ministry of Health of Turkey and HUNDD (2006) | Details are present in (7), including cites of Bach-Faig et al. (2011a) and Ministry of Health of Turkey and HUNDD (2006) | (7) |

**References**

1. Instituto de Información Estadística y Geográfica de Jalisco (IIEG). Población por entidad y municipios y edad desplegada según sexo (2015). https://iieg.gob.mx/general.php?id=4&idg=45 [Accessed May 2, 2019].

2. Hernández Sampieri Rm Fernández Collado C, Baptista Lucio MP. “Selección de la muestra”. In: Hernández Sampieri R, Fernández Collado C, Baptista Lucio MP. *Metodología de la investigación*. México: Editorial McGrawHill (2014) 6ª ed., p. 170-194.

3. Spiegel MR, Stephens LJ. Teoría Elemental del Muestreo. In: Spiegel MR, Stephens LJ, editors, *Estadística*. México: Mc Graw-Hill (2009) 4ª ed., p. 203-226.

4. Vanham D, Mak TN, Gawlik BM. Urban food consumption and associated water resources: The example of Dutch cities. Sci Total Environ (2016) 565. doi: https://doi.org/10.1016/j.scitotenv.2016.04.172

5. Vanham D. The water footprint of Austria for different diets. Water Sci Technol J Int Assoc Water Pollut Res (2013) 67:4. doi: 10.2166/wst.2012.623

6. Vanham D, Mekonnen MM, Hoekstra AY. The water footprint of the EU for different diets. Ecol Indic (2013) 32. doi: 10.1016/j.ecolind.2013.02.020

7. Vanham D, del Pozo S, Pekcan AG, Keinan-Boker L, Trichopoulou A, Gawlik BM. Water consumption related to different diets in Mediterranean cities. Sci Total Environ (2016) 573. doi: <https://doi.org/10.1016/j.scitotenv.2016.08.111>

8. Blas A, Garrido A, Willaarts BA. Evaluating the Water Footprint of the Mediterranean and American Diets. Water (2016) 8:10. doi:10.3390/w8100448

9. Germani A, Vitiello V, Giusti AM, Pinto A, Donini LM, del Balzo V. Environmental and economic sustainability of the Mediterranean Diet. Int J Food Sci Nutr (2014) 65:8. doi: https://doi.org/10.3109/09637486.2014.945152

10. Rosi A, Mena P, Pellegrini N, Turroni S, Neviani E, Ferrocino I, et al. Environmental impact of omnivorous, ovo-lacto-vegetarian, and vegan diet. Sci Rep (2017) 7:1. doi: 10.3389/fnut.2016.00040
